# Editorial: Perspectives in Primary Prevention Research for Breast Cancer: A Focus on Gene—Environment Interactions

**DOI:** 10.3389/fmed.2020.621959

**Published:** 2020-12-11

**Authors:** Sophie A. Lelièvre, Martine Bellanger, Victoria Seewaldt, Rabih S. Talhouk, Mary Beth Terry

**Affiliations:** ^1^Department of Basic Medical Sciences, Purdue University, West Lafayette, IN, United States; ^2^Purdue University Center for Cancer Research, West Lafayette, IN, United States; ^3^Institut de Cancérologie de L'Ouest, St Herblain, France; ^4^Department of Population Sciences, Beckman Research Institute, City of Hope, Pasadena, CA, United States; ^5^Department of Biological Sciences, American University of Beirut, Beirut, Lebanon; ^6^Department of Epidemiology, Columbia University, New York, NY, United States

**Keywords:** economy, communication, epidemiology, aging, tissue homeostasis, chemoprevention, epigenome, biomarker

## Introduction

We initiated the collaborative research program “international breast cancer & nutrition” (IBCN) in 2010 (1), responding to the increasing trends in breast cancer (BC) incidence globally (2–4). World-wide there are many similarities, including the increasing incidence of BC in young women, which demands more research on changing environmental exposures and transitions, such as increasing obesity, shifting diets and lower fertility. This special issue has been dedicated to what the IBCN considers at the heart of the problem, namely the interplay between BC susceptibility genes and the environment (5). The articles outlined below illustrate the importance of transdisciplinary approaches and networks and fall into four categories that warrant attention to reduce the global burden of disease: (1) lifestyle modifiers of risk; (2) early detection and risk reduction; (3) new avenues in research; and (4) economic benefits of global BC prevention.

## Lifestyle Modifiers of Risk

The global increase of BC is especially high in the Middle East, and Naja et al. comprehensively summarize reasons for the increase with a specific focus on nutrition and obesity. Their detailed review offers hope of possible reversal of the incidence trends, as many of the risk factors that they outline are modifiable. Complementing this article is a thorough review by Agurs-Collins et al. on the mechanisms and metabolic pathways with which changes in body fat and nutrition can affect BC risk. Considering biomarkers in future etiologic studies as well as potential targets for intervention studies are important perspectives highlighted by these authors. Key to understanding how to modify BC risk is to grasp that just like breast tumors are 3D, it is helpful to think about their causes as 3D. Forman presents a novel framework to understand BC trends and etiology through the 3D lens of (1) windows of susceptibility, (2) duration and intensity of exposures, and (3) pace of development and trajectories. All risk factors are affected by issues of timing, even one of the long-established risk factors for BC—age at menarche. Olsson and Olsson in an insightful mini-review remind us to think about the meaning of constructs and, rather than merely continuing to use age at menarche as a BC predictor, to focus more on menstrual activity (i.e., number of cycles before first pregnancy for premenopausal BC, and lifetime number of cycles for post-menopausal BC). This recommendation to focus on menstrual activity may be particularly useful for prediction as age at menarche is becoming more similar between countries, but there are still large gaps based on menstrual activity.

## Early Detection and Risk Reduction

There are recent technological advances and risk reduction efforts for BC. For example, Houghton et al. conducted a systematic review of BC management efforts that employ smartphone apps and identified two common themes of utilization, (1) clinical care coordination and (2) health care quality of life during and after a BC diagnosis. Moreover, an emerging interest in primary prevention is evidenced by apps that help predict BC risk and provide information related to primary BC prevention. There remain many opportunities to include for global use. The increase of BC incidence world-wide has spurred the need for cost-effective and minimally invasive early detection methods. Mammography screening is difficult to set up and less sensitive in “at high risk” young women with denser breasts. Nassar et al. discuss clinically easily accessible peripheral blood-based analysis of “liquid-biopsy.” Specifically, they evaluate emerging biomarker strategies that include circulating miRNA, proteins and nucleic acids, with methylation patterns for the latter, as well as exosomes that might augment routine screening tests. However, biomarkers will only be truly valuable if risk reduction can be implemented. An example of promising chemoprevention is low toxicity metformin, for which Jones et al. discuss some of the mechanisms of action. Noteworthy, their review emphasizes the importance of integrating the use of safe medications with other aspects of an intervention aimed to “make the whole person healthy.”

## New Avenues in Research

Focusing on mechanisms that control tissue homeostasis is a widespread approach to study risk factors and identify targets to inhibit cancer onset. This goal necessitates 3D cell culture models of phenotypically normal breast tissue, since normal breast biopsies are seldom accessible in most countries. The recent connection, using such models, between increased body mass and loss of epithelial polarity demonstrates how biology and epidemiology may be merged to identify markers of risk (6). Models that recapitulate breast polarity will be a useful resource for *in vitro* screening of modulators of risk. To ease the screening method, Manning et al. are presenting the radial profile analysis, an algorithm that objectively quantifies polarity in epithelial glandular structures from immunofluorescence images (available on the Open Science Framework).

Transposing tissue alterations measurable *in vitro* to the real organ for risk detection purposes is one of the many challenges of primary prevention. Building from the recent demonstration that tumor suppressor connexin-43 (Cx43) controls breast epithelial polarity and cell multilayering (7, 8), in their minireview Naser Al Deen et al. propose that Cx43-derived circRNA and associated sponged miRNA might be attractive liquid biopsy biomarkers indicative of Cx43 mRNA levels in tissues, hence serving as a signature axis for BC risk. Another area of excitement is magnetic resonance (MR) with which methodology progress made on breast tumors paves the way for potential risk assessment. Imaging with MR assesses breast density, the increase of which is an aggravating factor of cancer risk (9). Chhetri et al. provide an insightful discussion of MR methods available for the breast and on how techniques, like contrast enhanced perfusion MR imaging and MR spectroscopy, might be applied to detect microstructural and physiological alterations that are signs of increased risk for cancer. Tissue integrity via maintenance of homeostasis is also the topic covered by Fresques et al. who propose sustaining tissue organization by driving progenitor cells to terminally differentiate, or alternatively reducing or delaying the innate age-related immune changes in the breast that include chronic low-grade sterile inflammation, known as inflammaging. A case is made for repurposing warfarin and metformin for prevention, since both drugs act in part by modulating aging-associated changes at the tissue level.

The ultimate demonstration of sustained deleterious impact of risk factors on tissue homeostasis is an alteration of the epigenome. Duforestel et al. bring evidence that the pesticide/herbicide glyphosate specifically alters the expression of genes controlled by the epigenetic enzyme TET3 and synergizes with miR-182 (part of oxidative stress associated with aging tissues) to trigger mammary tumors. This first demonstration that a pollutant can synergize with a physiological alteration of cells is a powerful illustration of the concept of multifactorial disease relevant to cancer. The detection of DNA methylation changes characteristic of glyphosate in the blood opens new directions for epigenetic biomarkers to reveal potentially persistent effects of exposures.

## Economic Benefits of Global bc Prevention

The effects of prevention are measurable in the future despite the perceived concern about the current value of any action. Bellanger et al. provide evidence of the cost-effectiveness of lifestyle related interventions for BC. From a societal perspective physical activity programs are highly cost effective for BC and other major non-communicable diseases, and low-fat diet programs for post-menopausal women are cost-effective for breast and ovarian cancers. These encouraging findings on healthier lifestyle influence deserve attention from both individuals and public decision makers. However, the inherent link between people's environment and the epigenome requires urgent efforts in communication to better translate research on primary prevention to the public and policymakers, as clearly demonstrated by Perrault et al. Their core message is simple; researchers willing to advance their scientific knowledge have to be concomitantly willing to translate and disseminate their work to the public who will be able to act ultimately.

In sum, the articles in this issue highlight the importance of prevention to reverse the global rise in BC. In comparison to the large investment in BC therapies and detection, investment in primary prevention research has been much more limited. Prevention options like risk-reducing surgeries and chemoprevention for women at higher risk are restricted to certain countries and bring challenges like genetic testing and invasive interventions. In contrast, a focus on gene-environment interactions expands the perspectives in primary prevention research by adopting a holistic paradigm and promises a much wider population impact. The integration of environmental impact in health risk can be used extensively by policymakers while the world is currently facing many pandemics from COVID19 to climate change to widespread health inequities. A transdisciplinary approach through international collaborative networks like IBCN will be essential to continue to move forward and reduce the global burden of BC.

## Author Contributions

SL and MT have edited the different versions of the manuscript. All authors contributed to the writing of the manuscript.

## Conflict of Interest

The authors declare that the research was conducted in the absence of any commercial or financial relationships that could be construed as a potential conflict of interest.
